# Proline-Hydroxylated Hypoxia-Inducible Factor 1α (HIF-1α) Upregulation in Human Tumours

**DOI:** 10.1371/journal.pone.0088955

**Published:** 2014-02-12

**Authors:** Cameron E. Snell, Helen Turley, Alan McIntyre, Demin Li, Massimo Masiero, Christopher J. Schofield, Kevin C. Gatter, Adrian L. Harris, Francesco Pezzella

**Affiliations:** 1 Tumour Pathology Group, Nuffield Division of Clinical Laboratory Sciences, Radcliffe Department of Medicine, University of Oxford, Oxford, United Kingdom; 2 Molecular Oncology Laboratories, Department of Medical Oncology, Weatherall Institute of Molecular Medicine, University of Oxford, Oxford, United Kingdom; 3 Haemato-Oncology Group, Nuffield Division of Clinical Laboratory Sciences, Radcliffe Department of Medicine, University of Oxford, Oxford, United Kingdom; 4 Chemistry Research Laboratory, University of Oxford, Oxford, United Kingdom; Health Canada and University of Ottawa, Canada

## Abstract

The stabilisation of HIF-α is central to the transcriptional response of animals to hypoxia, regulating the expression of hundreds of genes including those involved in angiogenesis, metabolism and metastasis. HIF-α is degraded under normoxic conditions by proline hydroxylation, which allows for recognition and ubiquitination by the von-Hippel-Lindau (VHL) E3 ligase complex. The aim of our study was to investigate the posttranslational modification of HIF-1α in tumours, to assess whether there are additional mechanisms besides reduced hydroxylation leading to stability. To this end we optimised antibodies against the proline-hydroxylated forms of HIF-1α for use in formalin fixed paraffin embedded (FFPE) immunohistochemistry to assess effects in tumour cells *in vivo*. We found that HIF-1α proline-hydroxylated at both VHL binding sites (Pro402 and Pro564), was present in hypoxic regions of a wide range of tumours, tumour xenografts and in moderately hypoxic cells *in vitro*. Staining for hydroxylated HIF-1α can identify a subset of breast cancer patients with poorer prognosis and may be a better marker than total HIF-1α levels. The expression of unhydroxylated HIF-1α positively correlates with VHL in breast cancer suggesting that VHL may be rate-limiting for HIF degradation. Our conclusions are that the degradation of proline-hydroxylated HIF-1α may be rate-limited in tumours and therefore provides new insights into mechanisms of HIF upregulation. Persistence of proline-hydroxylated HIF-1α in perinecrotic areas suggests there is adequate oxygen to support prolyl hydroxylase domain (PHD) activity and proline-hydroxylated HIF-1α may be the predominant form associated with the poorer prognosis that higher levels of HIF-1α confer.

## Introduction

The activation of hypoxic signalling has a critical role in the pathogenesis of solid malignancies. Hypoxia-inducible factor 1 (HIF-1) consists of HIF-α and HIF-β subunits which heterodimerise and bind to discrete HIF-binding response elements in DNA, which mediate changes in transcription. In hypoxia, the increased activity of HIF-1 leads to the increased transcription of multiple genes [1] involved in genetic instability [2], angiogenesis [3], metabolism [4], pH regulation [5], invasion, metastasis, epithelial-mesenchymal transition [6] and radiation resistance [7]. The HIF transcriptional responses have been largely attributed to HIF-1α and HIF-2α, although there is a third HIF-α subunit, HIF-3α [8].

The HIF-α isoforms are normally efficiently degraded under normoxic conditions. This process firstly requires that HIF-1α is hydroxylated at one of two proline sites within two oxygen-dependent degradation domains (NODD Pro402, CODD Pro564), as catalysed by one or more of the three members of the prolyl hydroxylase domain (PHD) isoforms. Hydroxylation of either proline residue renders HIF-α susceptible to binding by the von Hippel-Lindau tumour suppressor (pVHL) E3 ligase complex, leading to ubiquitination and degradation by the proteasome [9]. In hypoxia, HIF-1α and/or HIF-2α accumulate. One cause of this accumulation is that the PHDs are inhibited in hypoxia [10]. In a second method of oxygen dependent regulation of HIF activity, an aspargine-residue in the *C*-terminal transactivation domain of HIF-α is hydroxylated by factor inhibitor HIF (FIH), a process that reduces binding of HIF to transcriptional coactivators.

Whether HIF-1α requires one or both proline sites hydroxylated *in vivo* to be efficiently degraded is not clear. Cells deficient in HIF-1α that are subsequently transfected with HIF-1α with either proline sites substituted within the *C*- and *N*-terminal ODD show approximately equal amounts of increased stability and reduced upregulation in hypoxia. If both proline sites are substituted, HIF-1α shows further increases in stability and no induction in hypoxia [11], [12]. The ODD domain of HIF-1α with both proline sites mutated fused to *E.coli* cytosine deaminase can still undergo VHL-dependent degradation suggesting additional mechanisms of regulation to those for which hydroxylation is essential [12]. The *C*- and *N*- terminal proline sites have different susceptibilities to hypoxia such that Pro402 hydroxylation is inhibited under higher oxygen tensions than Pro564 [13]. The observation of accumulated HIF-1α hydroxylated at Pro564 in cells under hypoxia suggests that VHL-mediated degradation may, at least under some contexts, be dependent on recognition of hydroxylation within the NODD [13].

In cell culture, inhibition of HIF-1α proline-hydroxylation requires levels of hypoxia below 1% oxygen [13], whilst the accumulation of HIF-1α is seen to exponentially rise below 6% oxygen [14]. The kinetic properties of the PHD, in particular PHD2, are proposed to be related to their roles as oxygen sensors, and it is notable that PHD2 has an apparently slow reaction with oxygen [15]. However, the K_m_ (oxygen) for PHD activity using recombinant enzymes is well above (100 µM) the 10–30 µM oxygen levels typically available to tissues [10], [16]. Although in cells it is the overall flux through the pathways that is important, this observation suggests that PHD-independent mechanisms for HIF-α degradation may be relevant.

Modulators of PHD activity, beside oxygen, have been described; these may account, at least in part, for the disparities between the biochemical properties of the PHDs and the observed HIF-1α levels. These modulators include mitochondrial derived reactive oxygen species (ROS) [4], [17], elevated levels of Krebs cycle intermediates such as succinate and fumarate [18], [19], iron and ascorbate [20]. Although these factors can affect hydroxylation of HIF-1α, whether they modulate the degrading activity of the VHL-containing complex is not clear. Other factors can also affect the amount of HIF-1α available for transcription. These include increased HIF-1α production by activated mTOR signalling [21] and VHL-independent degradation by heat shock protein 70 (Hsp70) and carboxyl terminus of HSP-interaction protein (CHIP) [22].

In this study, we aimed to determine if proline-hydroxylated HIF-1α formed a component of upregulated HIF-1α expressed *in vivo* in tumours. As HIF-1α in reoxygenated tissue has a half-life of less than 1 minute [23], we would not expect to see this unless there were other factors that regulate degradation.

In order to assess expression of proline-hydroxylated HIF-1α, we have validated commercially available hydroxylation-specific antibodies against the two proline-hydroxylation sites in HIF-1α for use in FFPE immunohistochemistry. These antibodies have previously been validated for their use in western blotting [13]. We aimed to validate and use these antibodies for use in immunohistochemistry to gain insights into the hydroxylation status, and therefore mechanism, of HIF-1α stabilisation both *in vitro* models of hypoxia as well as in tumours *in vivo*.

## Materials and Methods

### Ethics statement

The use of de-identified patient tissue was granted by the Oxford Radcliffe Biobank Access Committee. The Oxford Radcliffe Biobank (http://orb.ndcls.ox.ac.uk/) obtained written consent for the use of these samples in research. The Oxford Radcliffe Biobank has ethical approval to collect tissue and clinical information from patients granted by the NHS Health Research Authority on the advice of Oxfordshire Regional Ethical Committee C (09/H0606/5 accessible at http://www.nres.nhs.uk/research-tissue-bank-summaries/?entryid70=131338&p=8).

### Cells and culturing conditions

MCF7, 786-O, RCC4, U87, HCT-116 and MDA-MB-468 cells were cultured in DMEM (Lonza) supplemented with 10% foetal calf serum (Life Technologies) and 2 mM L-glutamine. RCC4 cells stably expressing a wild-type VHL (RCC+VHL) or empty vector (RCC-VHL) and 786-O cells were a gift from W. Kaelin [24]. MCF7, U87, HCT-116 and MDA-MB-468 cells were acquired from the ATCC. MCF7 cells were treated with 1 mM dimethloxalylglcine (DMOG) (Sigma-Aldrich), 10 mM of the proteasome inhibitor MG-132 (Millipore) or DMSO alone for 4 hours. Tumour spheroids were created from cell lines by plating cells in non-adherent round-bottomed 96-well dishes (Corning) and centrifuging at 2000×G for 10 minutes.

### Cell pellet preparation

Cells were trypsinised, pelleted and washed, prior to resuspension in 10% neutral-buffered formalin for 24 hours. The formalin was removed and cells were then resuspended in 2% molten agarose dissolved in 10% formalin. The cells and agarose suspension was then centrifuged in a 1.5 ml tube and transferred to ice to form a cell pellet. The pellet was removed from the tube and placed within a cassette for overnight processing, after which, the pellet was wax embedded and sectioned.

### Plasmid mutagenesis and transfection

The full-length HIF-1α expression vector was purchased from OriGene. The P402A mutation was produced by using the Quikchange site-directed mutagenesis kit XL (Stratagene) and the primers CTCCAGCGGCTGCGGCCAGCAAAGT (Forward) and ACTTTGCTGGCCGCAGCCGCTGGAG (Reverse). The P564G mutation was generated in the same way using the primers TAGACTTGGAGATGTTAGCTGGCTATAGCCCAATGGATGATG (Forward) and CATCATCCATTGGGATATAGCCAGCTAACATCTCCACGTCTA (Reverse). Primers were purchased from Eurogentec. Cells were transfected using Turbofect (Thermo Scientific) according to manufacturers protocol.

### Mouse xenografts

Mice were house at the Biomedical Sciences department (University of Oxford) and all procedures were carried out under a Home Office license. Cells were injected into the flank of BALB/c *nu/nu* mice subcutaneously. Mice were sacrificed when tumours reached 1.44 cm^3^ by cervical dislocation. Tumours were excised, fixed in 10% formalin overnight, cut-up and processed in the same way as resected human diagnostic tissue.

### Antibodies

The antibody against HIF-1α, insensitive to hydroxylation was a mouse monoclonal from BD Biosciences (Catalogue Number 610958). Hydroxylation-specific HIF-1α antibodies were raised in rabbit; P402 was from Millipore (07-1585) and P564 from Cell Signaling Technologies (3434S). Antibodies against PHD-1, PHD-2 and PHD-3 were produced and characterised by this group previously [25]. The antibody against VHL for immunohistochemistry was a mouse monoclonal from BD Biosciences (Catalogue Number 556347) and for western blotting was from Cell Signaling Technologies (2738S).

### Immunohistochemistry

4 µM sections of cells and tissue were deparaffinised and antigen retrieved in Target Retrieval Solution (S1699, Dako) using a Biocare decloaking chamber. Sections were blocked with 2.5% normal horse serum for 30 minutes and then incubated with primary antibody diluted in RPMI overnight at 4°C. Dilutions for primary antibodies used were: anti-HIF-1α total 1∶100; anti-hydroxylated HIF-1α P402 1∶1000; anti-hydroxylated HIF-1α P564 1∶800; anti-VHL 1∶500. Bound antibody was detected using the Novalink Polymer Detection System (Leica) for mouse antibodies and the two-step rabbit HRP polymer (Menarini Diagnostics) for rabbit antibodies, counterstained with haematoxylin. Conditions for PHD staining were as previously described[25]. Staining was independently scored by two pathologists (CS and FP) and discrepancies were resolved by consensus. Total and proline-hydroxylated HIF-1α antibody staining was scored as being either present or absent, while PHD1, 2, 3 and VHL staining was scored on a semiquantitative score from 0–3.

### Patient Material

Formalin-fixed paraffin-embedded (FFPE) tumour tissue from the hypoxic regions of 109 patients with head and neck cancer, 141 patients with breast cancer, 20 patients with lung cancer and 47 patients with bladder cancer were stained by immunohistochemistry using tissue microarrays (TMAs) as described previously [26].

For patients with breast cancer, FFPE tissues were obtained for 287 sequential patients with breast carcinoma who underwent surgery between 1989 and 1998 at the John Radcliffe Hospital, Oxford. The number of patient samples stained by immunohistochemistry with all the antibodies was limited by core exhaustion and sporadic section loss. Patients had been treated by wide local excision and postoperative radiotherapy, or by mastectomy with or without postoperative radiotherapy. Postoperative chemotherapy (600 mg/m^2^ cyclophosphamide, 40 mg/m^2^ methotrexate and 600 mg/m^2^ 5-fluorouracil IV on day 1 of a 21 day cycle, repeated for six cycles) and hormonal therapy were offered according to local protocols. The sample size was determined by the availability of tissue with clinical follow-up data and ethical approval. Two cases had no clinical follow up. Follow-up data was correct as of January 2008. The median follow-up time was 10 years, with a medial overall survival of 14.5 years.

### Statistical analysis

Kaplan-Meier curves were assembled in Prism version 4 (GraphPad software). Mean survival times were estimated from Kaplan-Meier curves. Comparisons between survival curves were made by using the logrank test. Correlations between immunohistochemical scores were calculated using Pearson's correlation co-efficient and significance assessed using with Student's t-test. All calculations were performed using SPSS version 21 (IBM).

### Immunoblots

Cells were lysed on the bench (for normoxic samples) or within the hypoxic chamber (for hypoxic samples) with equilibrated Complete Lysis-M with protease inhibitors (Roche) and quantified using the BCA assay kit (Pierce). Protein was separated using Novex Bis-Tris 4–12% gels (Life technologies) and transferred to nitrocellulose membrane. Membranes were blocked with 5% non-fat milk powder dissolved in PBS-T, and incubated with primary antibodies overnight. Densitometric analysis was performed using ImageJ [27].

### Cell line sequencing data

The data was obtained from the Wellcome Trust Sanger Institute Cancer Genome Project web site, http://www.sanger.ac.uk/genetics/CGP.

## Results

### HIF-1α proline hydroxylation in cancer cell lines in hypoxia

**Figure 1:**
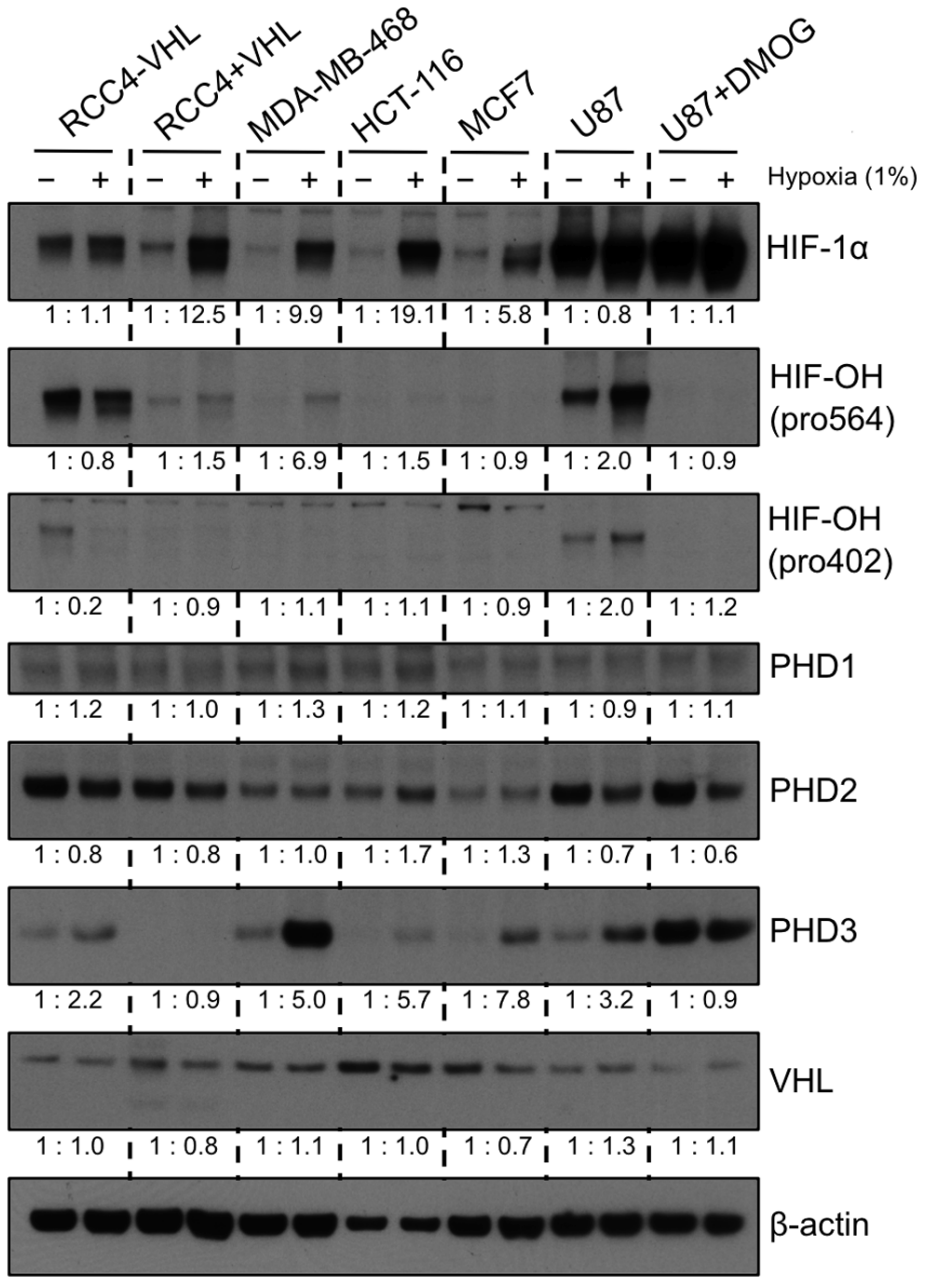
Cell lines show a heterogenous pattern of expression of proline-hydroxylated HIF-1α in moderate (1%) hypoxia. A panel of cell lines were incubated in 1% hypoxia for 16 hours, probed with the indicated antibodies by western blot. A lack of immunoreactivity for U87 cells treated with DMOG demonstrate that hydroxylated HIF-1α antibodies do not show cross-reactivity for the unhydroxylated form. The ratios indicate the actin-normalised change in expression between normoxia and hypoxia, estimated by densitometry (to one decimal place).

Initially, we tested a panel of cell lines to determine whether cells *in vitro* showed increased quantities of proline-hydroxylated HIF-1α at physiologically relevant levels of hypoxia. Cells were grown and lysed within a hypoxic chamber at 1% atmospheric oxygen using equilibrated buffers, and HIF-1α proline hydroxylation was determined using antibodies specific for each proline-hydroxylation site (Figure 1.) [13].

We found that at 1% oxygen, there was a diverse pattern of proline-hydroxylation of HIF-1α between different cell lines. At these conditions, RCC4 cells, deficient of VHL activity (RCC4-VHL) (which still have a low level of expression [28]), showed inhibition of hydroxylation at Pro402 (0.2 of normoxic levels), but only slightly at Pro564 (0.8 of normoxic levels). RCC4 cells with active VHL (RCC4+VHL) demonstrated robust induction of total HIF-1α in hypoxia, with a 1.5 fold increase in the hydroxylated form at Pro564 and minimal changes at Pro402. MDA-MB-468 cells showed increased hydroxylated HIF-1α just at Pro564, whereas HCT-116 showed large increases in total HIF-1α, which was not accompanied by increases in proline hydroxylation at either site. MCF7 cells showed an increase in total HIF-1α in hypoxia that was accompanied by minimal changes in the amount of proline-hydroxylated HIF-1α at Pro564 and Pro402. U87 cells showed increased proline-hydroxylated HIF-1α at both Pro564 and Pro402 (both 2.0 fold increases), and a slight decrease in total HIF-1α. U87 cells treated with DMOG were included to demonstrate that our hydroxylated HIF-1α antibodies did not show cross-reactivity to the unhydroxylated form.

We assessed whether the differences in induction in hydroxylated HIF-1α were associated with levels of the PHD enzymes or VHL. Consistent with previous reports, PHD1 was expressed at very low levels [29]. PHD2 levels were mildly induced by hypoxia in HCT-116 and MCF7 cells. PHD3 was strongly upregulated by hypoxia in all cell lines (apart from RCC4+VHL). VHL levels were not significantly altered in hypoxia. No correlation between the pattern of hydroxylated HIF-1α accumulation and levels of PHD enzymes or VHL was identified.

### Validation of proline-hydroxylated specific HIF-1α antibodies

**Figure 2:**
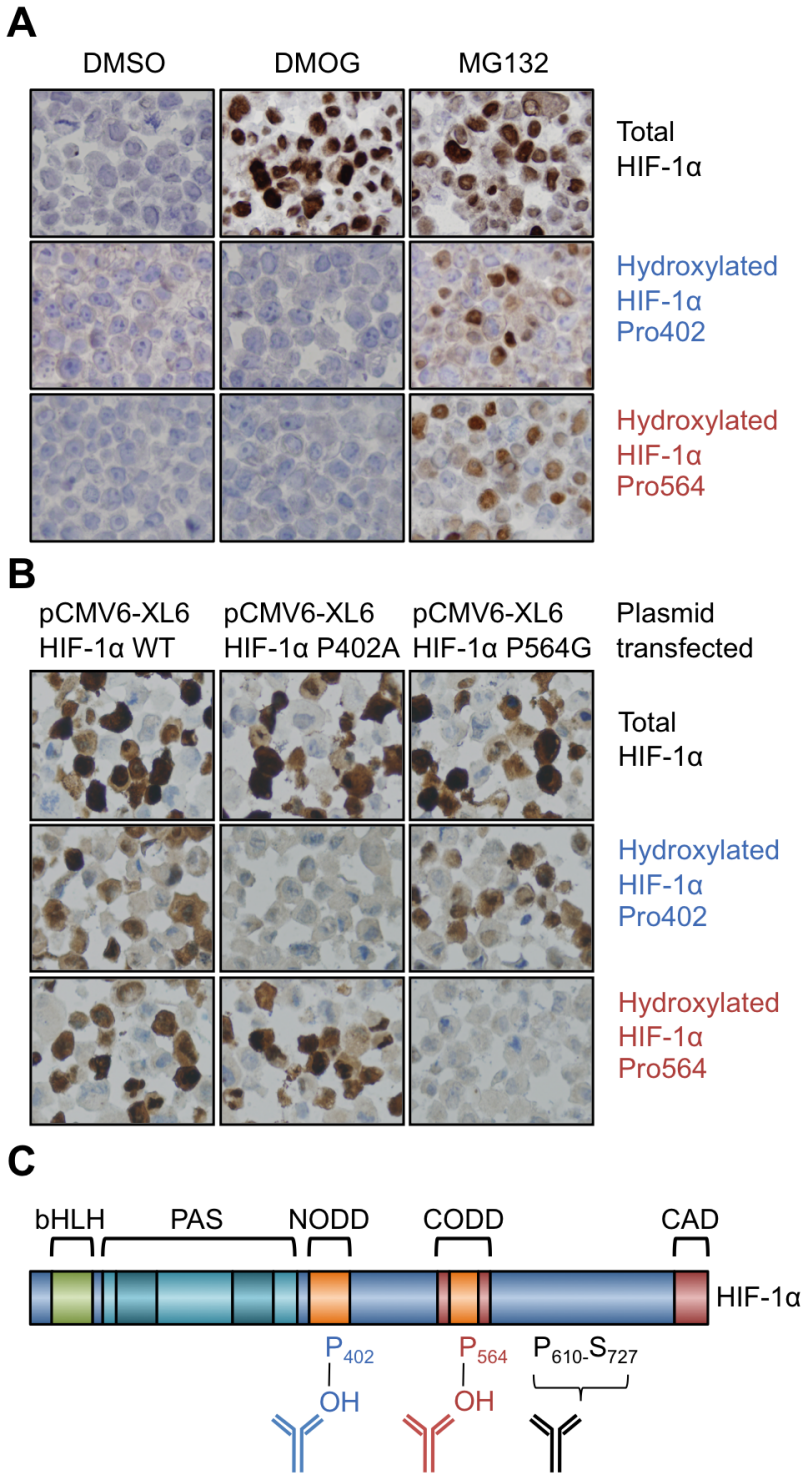
Validation of proline-hydroxylated HIF-1α specific antibodies on formalin-fixed paraffin embedded cell pellets. **A.** MCF7 cells treated with vehicle (DMSO), DMOG or MG132 and probed with the indicated antibodies. **B.** 786-o cells transfected with either HIF-1α WT, P402A or P564G and probed with the indicated antibodies. **C.** Domain structure of HIF-1α and antibody binding regions. The figure highlights: basic helix-loop-helix (bHLH) domains; PAS domain; the amino-terminal oxygen-dependent degradation domain (NODD) the carboxy-terminal oxygen-dependent degradation domain (CODD) and the carboxy-terminal activation domain (CAD).

In order to determine that HIF-1α antibodies were specific for the hydroxylated form for use in immunohistochemistry, we treated MCF7 cells with a cell-penetrating prodrug form of a 2-oxoglutarate oxygenase/PHD inhibitor (dimethyloxalylglycine, DMOG) and the proteasomal inhibitor MG132. The cells were fixed and processed in the same way as tissue samples. The hydroxylated-specific antibodies only recognised HIF-1α that accumulated with proteasomal inhibition, and not that which accumulated with PHD inhibition, whereas the total HIF-1α antibody recognised the hydroxylated and non-hydroxylated forms (Figure 2A.). It can be assumed that the HIF-1α that accumulated with MG-132 treatment was entirely hydroxylated (since this modification is the target for the ubiquitinating complex with VHL), however the staining with the hydroxylated-specific antibodies was weaker than the total HIF-1α antibody. This indicates that the hydroxylated HIF-1α antibodies are less sensitive than the total HIF-1α antibody.

To investigate whether each hydroxy-specific antibody was specific for the individual prolines in HIF-1α, we used 786-o cells that are defective in VHL and have a HIF-1α homozygous deletion. We transfected into these cells a full length HIF-1α construct, one with a mutated proline 402 (P402A) and another with mutated proline 564 (P564G). The hydroxy-specific antibodies did not bind when their respective proline sites were mutated (Figure 2B.). Mutation of either proline did not affect the hydroxylation of the other proline or the ability of these to be detected. 786-o cells have high levels of HIF-2α [24] and the lack of staining with hydroxy-specific antibodies in cells transfected with mutant plasmids shows there is no appreciable cross-reactivity to hydroxylated HIF-2α. The binding location of the total HIF-1α antibody is between proline 610 and serine 727. The location of antibody binding in relation to HIF-1α domains is illustrated (Figure 2C.).

### Expression of proline-hydroxylated HIF-1α in tumour models

**Figure 3:**
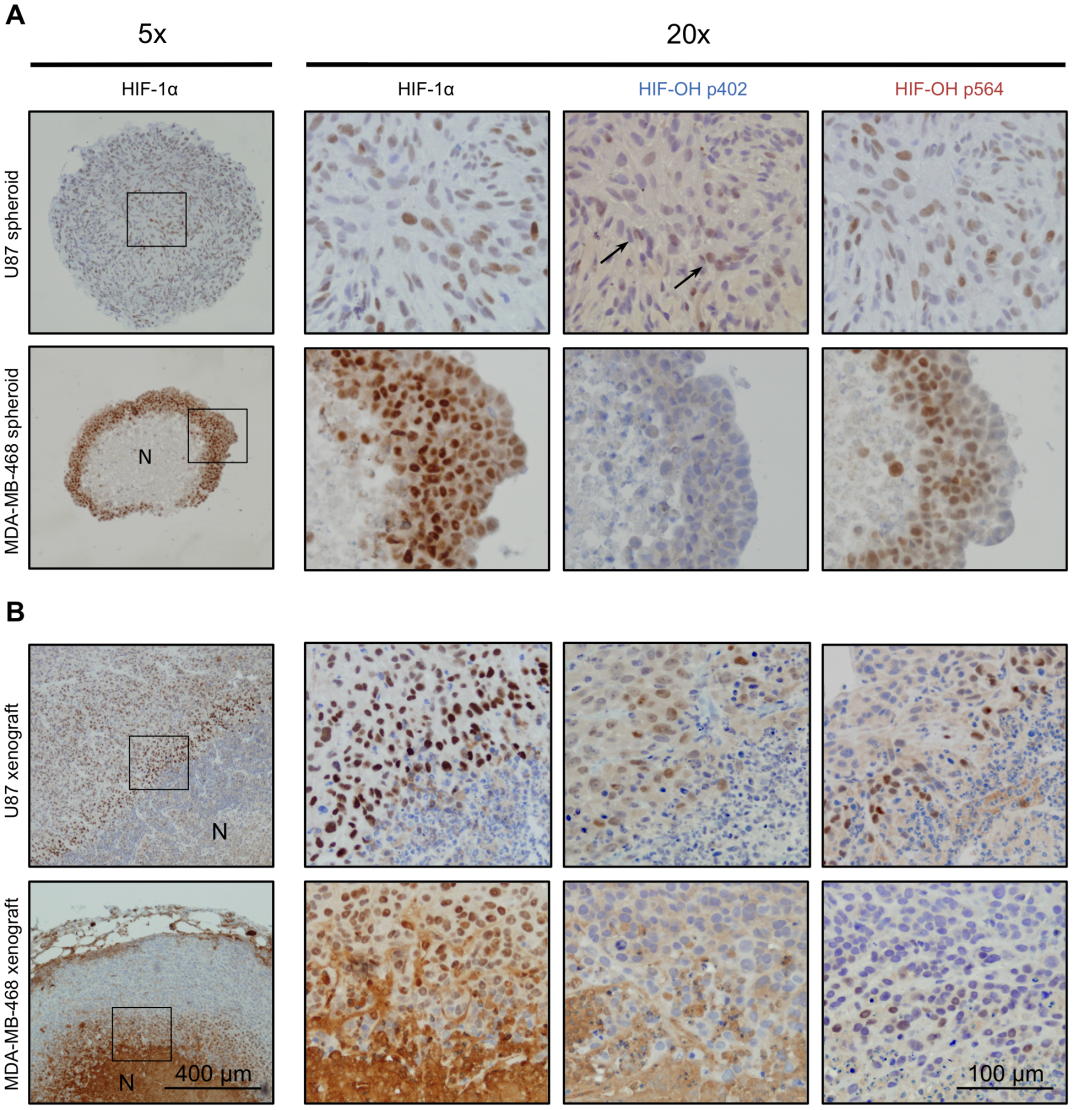
The expression of proline-hydroxylated and total HIF-1α in tumour models. Top two rows: U87 and MDA-MB-468 tumour spheroids. Left-most panel is a low power view (5× magnification) of spheroid stained with total HIF-1α. Box indicates area of enlargement for next three images. Right three images are high (20X) power serial sections stained with the indicated antibodies. Arrows indicate subtle positive nuclear staining. Bottom two rows: U87 and MDA-MB-468 mouse tumour xenografts. N =  necrosis. Scale bars apply to all images of the same magnification.

Having established the specificity of hydroxylated HIF-1α antibodies, we determined the expression *in vitro* and *in vivo* in tumour models. Two cell lines (U87 and MDA-MB-468) derived from glioblastoma and breast adenocarcinoma were used and both have wild-type VHL as determined by sequencing. In tumour spheroids, U87 cells showed an accumulation of HIF-1α substantially hydroxylated at both Pro402 and Pro564 (Figure 3.). MDA-MB-468 cells show a greater degree of central necrosis when cultured as spheroids. There was significant accumulation of hydroxylated HIF-1α at Pro564 in MDA-MB-468 spheroids, however hydroxylation at Pro402 was not detected. The hydroxylation at Pro564 extended up to the necrotic core of the spheroid.

In U87 xenografts, hydroxylated HIF-1α at Pro402 and Pro564 showed increasing staining towards the necrotic areas, similar in pattern to that of total HIF-1α (Figure 3.). Cells that juxtapose necrosis show the strongest hydroxylated HIF-1α staining at both proline hydroxylation sites. The intensity of staining is less for the hydroxylated HIF-1α antibodies, consistent with being less sensitive than total HIF-1α staining. Tumour xenografts created from MDA-MB-468 cells showed the same pattern as that seen in spheroids; there was increased hydroxylated HIF-1α at Pro564, but none detectable at Pro402. The hydroxylated HIF-1α was seen extending up to the viable cells that juxtaposed the necrotic area.

### Expression of hydroxylated HIF-1α in hypoxic human tumours

**Figure 4:**
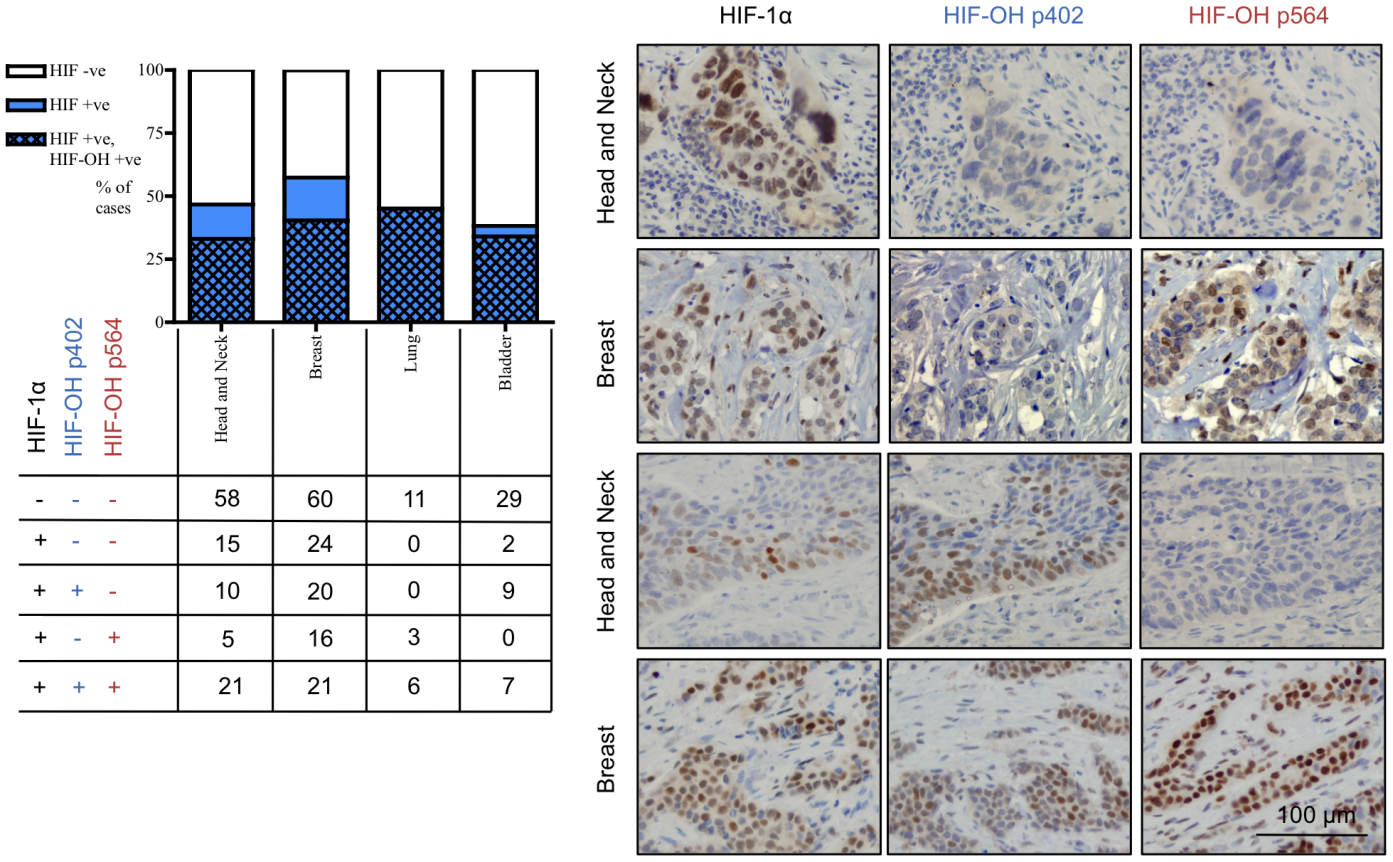
Proline-hydroxylated HIF-1α is present in a high proportion and wide range of tumours. Graph indicates relative proportion of HIF-1α positive tumours, and the proportion of which also show detectable staining for proline-hydroxylated HIF-1α. Numbers below represent numbers of cases with that particular staining pattern. Photomicrographs representative of cases showing different patterns of expression of proline-hydroxylated HIF-1α. Scale bar (bottom right) applies to all images.

Having determined variability in the expression of proline-hydroxylated HIF-1α in VHL competent cell lines, we used a panel of tissue microarrays to evaluate the expression of total and proline-hydroxylated HIF-1α in hypoxic head and neck, breast, lung and bladder carcinomas (Figure 4.). Overall, 50% of tumours had detectable HIF-1α (47% head and neck, 57% breast, 45% lung and 38% bladder). 74% of tumours evaluated that had detectable HIF-1α, also had proline-hydroxylated HIF-1α (71% head and neck, 70% breast, 100% lung and 89% bladder). Of the cases that had only one hydroxylation site detectable, 62% were hydroxylated just at proline 402 and 38% just at proline 564, within limits of detection. The different patterns of proline-hydroxylated HIF-1α in tumours are represented in Figure 4.

### Expression of proline-hydroxylated HIF-1α predicts adverse prognosis in breast cancer

**Figure 5:**
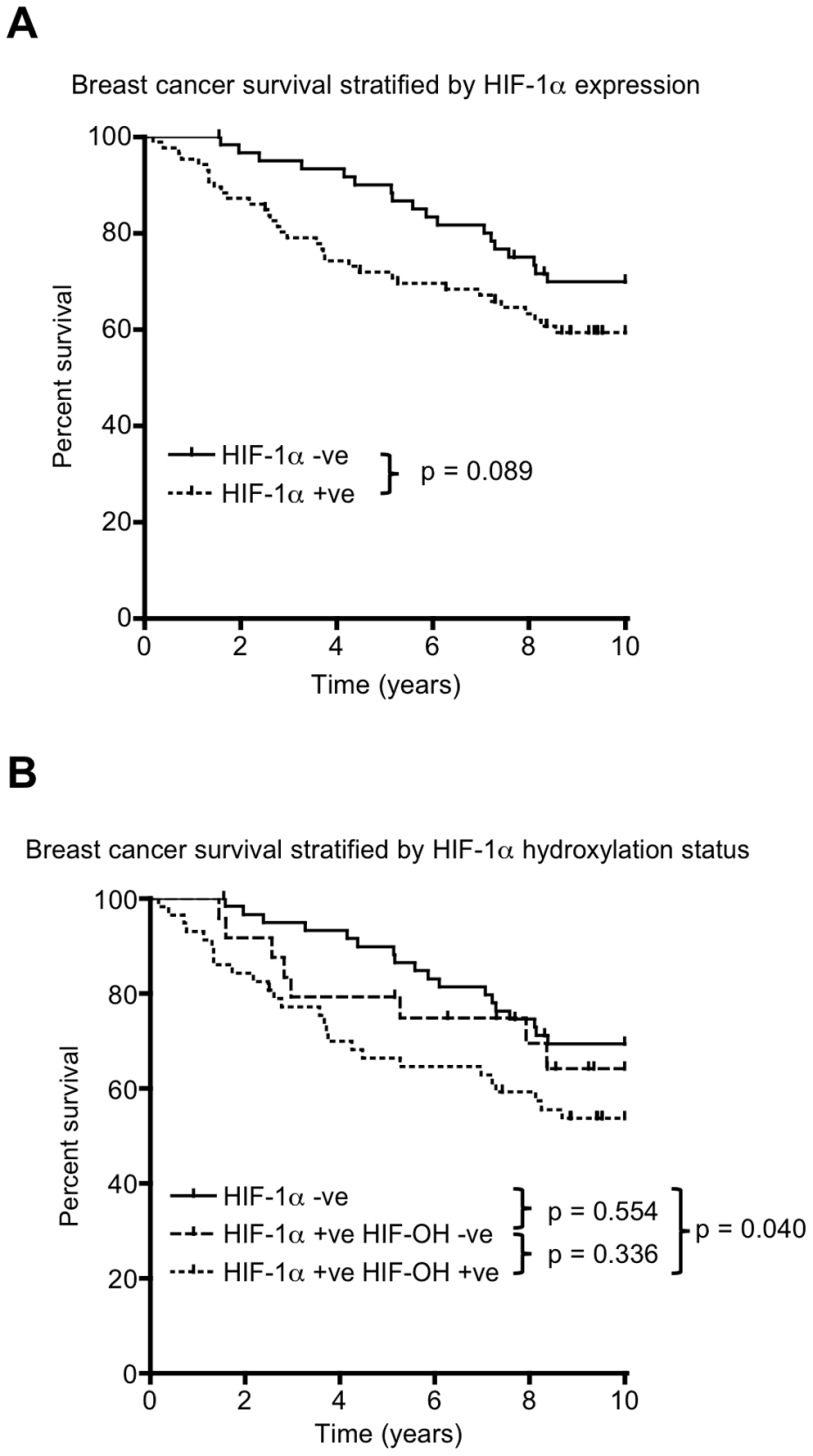
Expression of proline-hydroxylated HIF-1α predicts poorer overall survival in primary breast cancer. **A.** Kaplan-Meier curve for overall survival comparing patients with tumours that are HIF-1α+ve or –ve. **B.** Kaplan-Meier curves comparing patients with tumours that are negative for HIF-1α, HIF-1α+ve but hydroxylated HIF-1α –ve and HIF-1α+ve and hydroxylated HIF-1α+ve. P values quoted are the result of the logrank test for comparison of survival curves. Further information concerning censored cases and survival times is presented in Supporting Tables S1–4 in File S1.

We evaluated whether proline-hydroxylated HIF-1α was prognostic in primary breast cancer. In this cohort, 86/147 patients had detectable HIF-1α in their primary resected tumours. The expression of HIF-1α was associated with shorter overall survival although this was not significant (8.65 vs 7.43 years, p = 0.089) (Figure 5A and Supporting Tables S1 and S2 in File S1.). 141 of these patients had material available to assess for proline-hydroxylated HIF-1α. When these patients were stratified into those with tumours containing detectable proline-hydroxylated HIF-1α versus those with HIF-1α without hydroxylation, patients with primary tumours containing hydroxylated HIF-1α had significantly worse overall survival than patients with tumours without detectable HIF-1α (7.06 vs. 8.63 years, p = 0.040) (Figure 5B and Supporting Tables S3 and S4 in File S1). No significant differences in survival were observed between patients with HIF-1α positive tumours, comparing hydroxylated and hydroxylation-negative HIF-1α.

### The correlation of HIF-1α hydroxylation status with PHD enzymes and VHL

We stained and scored expression of the PHD enzymes (1–3) and VHL and evaluated whether levels of these correlated with expression of total, proline-hydroxylated, or unhydroxylated HIF-1α (Table 1). We found, as expected, HIF-1α and hydroxylated HIF-1α positivity correlated. The levels of PHD1 correlated with PHD2, and the levels of PHD2 and PHD3 also correlated. The levels of VHL significantly correlated with the expression of HIF-1α in cases that did not show detectable proline-hydroxylated HIF-1α (unhydroxylated HIF-1α) (*r* = 0.201, p = 0.030).

**Table 1:** Correlations between total, proline-hydroxylation and unhydroxylated HIF-1α and enzymes involved in HIF-1α degradation.

Pearson correlation *p* value	HIF-1α Pro402	HIF-1α Pro564	HydroxylatedHIF-1α	UnhydroxylatedHIF-1α	PHD1	PHD2	PHD3	VHL
HIF-1α	**<0.0001** a	**<0.0001** a	**<0.0001** a	**<0.0001** a	0.857	0.621	0.914	0.067
HIF-1α Pro402		**<0.0001** a	**<0.0001** a	**0.030** b	0.140	0.830	0.695	0.766
HIF-1α Pro564			**<0.0001** a	**<0.0001** b	0.181	0.604	0.607	0.935
Hydroxylated HIF-1α				**<0.0001** b	0.105	0.878	0.796	0.962
Unhydroxylated HIF-1α					0.085	0.785	0.545	**0.030** a
PHD1						**0.001** a	0.052	**0.034** a
PHD2							**<0.0001** a	0.100
PHD3								0.511
VHL								

a significant positive correlation;

b significant negative correlation.

The expression of PHD enzymes 1, 2 and 3 and VHL were scored and the expression correlated with HIF hydroxylation pattern using Pearson's correlation coefficient. Significant associations are in bold.

## Discussion

Overall, whilst the results are not fully quantitative, our observations on the physiology of stabilised HIF-1α in tumours and cancer cell lines reveal that a substantial component of stabilised HIF-1α in tumours is proline-hydroxylated, and in many cases is seen hydroxylated at both potential proline-hydroxylation sites within the ODD. Data from both cells lines and tumour xenograft models extends this observation demonstrating conclusively that accumulation of proline-hydroxylated HIF-1α occurs in tumours and cell lines that express wild-type VHL. The large numbers and wide range of tumours that express proline-hydroxylated HIF-1α suggest that the increased levels of hydroxylated HIF-1α, which is likely transcriptionally active, is an important part of the physiological hypoxia sensing mechanism in moderate levels of hypoxia. It is possible that the transcriptional activity of differently proline-hydroxylated HIF-1α varies, possibly as a consequence of altered protein-protein interactions involving pVHL, as is the case for CAD-hydroxylation, which reduces binding of HIF-α to coactivator proteins.

In cell lines exposed to hypoxia we observe heterogeneity in the accumulation of proline-hydroxylated HIF-1α, suggesting that tumour cells have major differences in their degradation pathways. There is thus clearly another mechanism of HIF regulation that occurs post proline-hydroxylation which is important in tumours and which is differentially exploited by different cell lines. Importantly, the level of VHL or PHD enzymes does not correlate with the propensity of cells to accumulate hydroxylated HIF in hypoxia. The stabilisation of hydroxylated HIF-1α at Pro564 in normoxia has been described in cells expressing a constitutively active Akt [30], and interestingly both U87 and MDA-MB-468 cells have homozygous mutations in PTEN, a negative regulator of the Akt pathway. Furthermore, hypoxia activates Akt at moderate levels of hypoxia (5% oxygen and below) [31]. HCT116 and MCF7 cells, which do not show increases in hydroxylated HIF-1α in hypoxia, have wild type PTEN. This suggests that activation of Akt versus other oncogenically activated pathways may potentially contribute towards the differential responses observed.

*In vivo*, we observed staining for proline-hydroxylated HIF-1α, even in the most hypoxic regions of MDA-MB-468 and U87 xenografts, demonstrating hypoxic inhibition of hydroxylated HIF-1α degradation *in vivo*. This staining extended up to the areas of necrosis, suggesting that the oxygen levels were sufficient to support HIF hydroxylation in these tumours. We were able to detect HIF-1α proline-hydroxylation in 79% of human tumours expressing HIF-1α. In the other 21%, tumours had HIF-1α yet no detectable hydroxylated HIF-1α. The data supports the notion that a substantial proportion of HIF-1α in the majority of tumours is proline-hydroxylated and that hypoxia can cause the degradation of proline-hydroxylated HIF-1α to be rate limiting.

Our finding that hydroxylated HIF-1α in primary breast cancer is associated with significantly poorer survival (unlike total HIF-1α) suggests that a more moderate level of hypoxia that allows for continued HIF-1α hydroxylation may favour malignant progression. Tumours are able to stabilise the proline-hydroxylated form of HIF-1α, providing another mechanism for regulating HIF in cancer. The activation of oncogenes associated with the stabilisation of hydroxylated HIF-1α may alternatively contribute to poor outcome. The expression of proline-hydroxylated and non-hydroxylated HIF-1α may explain why some studies fail to find a correlation between high HIF-1α tumour levels and adverse clinical outcome [32], [33]. The presence of proline-hydroxylated HIF-1α may also explain why there is a lack of correlation in tumours between HIF-1α levels and pO_2_ levels [34], [35].

A previous study detected an increase in proline-hydroxylated HIF-1α under hypoxia *in vitro*, in the tumour cell lines HeLa and HT1080 at Pro564 using similar methodology [13]. We found a number of tumours (and the MDA-MB-468 cell line) in which we only detected proline-hydroxylated HIF-1α at one site, although this was not universally at proline site 402 or 564. In tumours where proline-hydroxylated HIF-1α is detected, 29% are mono-hydroxylated at Pro402, 20% at Pro564 and 51% of tumours are proline-hydroxylated at both sites, highlighting biological heterogeneity between tumours.

The underlying mechanism(s) leading to the accumulation of proline-hydroxylated HIF-1α in tumours is unknown. The identification of these mechanisms is important because they may present new therapeutic possibilities by targeting increased HIF-1α degradation. Recent evidence suggests that overexpression of the oncogene c-Myc can also cause the accumulation of proline-hydroxylated HIF-1α in cell lines by decreasing the interaction of VHL with HIF-1α [36]. Post-translational modification of VHL, such as SUMOylation may cause oligomerisation and reduce its ability to recognise and degrade HIF-1α in hypoxia [37]. We found that higher levels of VHL were associated with the presence of HIF-1α, without detectable proline hydroxylation, supporting the possibility that high levels of VHL may prevent the accumulation of hydroxylated HIF-1α.

The PHD isoforms differentially hydroxylate HIF-1α. In cell lines, PHD2 is the most abundant HIF prolyl hydroxylase, and performs a non-redundant role in HIF degradation [29], [38]. PHD3 preferentially hydroxylates Pro564 (CODD), while PHD1 and 2 can hydroxylate both NODD and CODD sites, although Pro564 is hydroxylated prior to Pro402 [12], [38]. The regulation is complicated by the hypoxic induction of PHD2 and PHD3, and additional mechanisms to promote HIF accumulation may be required to prevent complete HIF degradation in the chronic hypoxic conditions found in tumours [29]. Our analysis of PHDs found no relationship to levels of proline-hydroxylated HIF-1α consistent with post-hydroxylation regulation.

In conclusion, we have demonstrated that a significant proportion of HIF-1α that accumulates in hypoxia *in vitro* and *in vivo* is proline-hydroxylated. This is seen in tumours that do not have known VHL mutations, and this represents a novel mechanism of HIF-1α accumulation. Determining the pathways by which the degradation of proline-hydroxylated HIF-1α is blocked may allow for targeted therapy to reverse this adaptation and reduce the tumourigenic phenotypes that are associated with higher levels of HIF signalling.

## Supporting Information

File S1 Table S1. Cases and events of patients with breast cancer, stratified by HIF-1α positivity. The numbers (N) of patients stratified by HIF-1α positivity, including those that had censored observations, used in the construction of Kaplan Meier curves in Figure 5A. Table S2. Mean survival of patients with breast cancer, stratified by HIF-1α positivity. The mean survival of patients with breast cancer stratified by HIF-1α positivity as estimated from Kaplan-Meier curves in Figure 5A. The standard error and 95% confidence interval for these estimates is included. Table S3. Cases and events of patients with breast cancer, stratified by HIF-1α hydroxylation status. The numbers (N) of patients stratified by HIF-1α hydroxylation status, including those that had censored observations, used in the construction of Kaplan Meier curves in Figure 5B. Table S4. Mean survival of patients with breast cancer, stratified by HIF-1α hydroxylation status.The mean survival of patients with breast cancer stratified by HIF-1α hydroxylation status as estimated from Kaplan-Meier curves in Figure 5B. The standard error and 95% confidence interval for these estimates is included.
(DOCX)
