# Supplementary material for: Proline-Hydroxylated Hypoxia-Inducible Factor 1α (HIF-1α) Upregulation in Human Tumours
Source: PLoS One. 2014 Feb 12;9(2):e88955. doi: 10.1371/journal.pone.0088955 (PMC3923075; doi:10.1371/journal.pone.0088955)
Supplement: File S1 — Table S1. Cases and events of patients with breast cancer, stratified by HIF-1α positivity. The numbers (N) of patients stratified by HIF-1α positivity, including those that had censored observations, used in the construction of Kaplan Meier curves in Figure 5A. Table S2. Mean survival of patients with breast cancer, stratified by HIF-1α positivity. The mean survival of patients with breast cancer stratified by HIF-1α positivity as estimated from Kaplan-Meier curves in Figure 5A. The standard error and 95% confidence interval for these estimates is included. Table S3. Cases and events of patients with breast cancer, stratified by HIF-1α hydroxylation status. The numbers (N) of patients stratified by HIF-1α hydroxylation status, including those that had censored observations, used in the construction of Kaplan Meier curves in Figure 5B. Table S4. Mean survival of patients with breast cancer, stratified by HIF-1α hydroxylation status.The mean survival of patients with breast cancer stratified by HIF-1α hydroxylation status as estimated from Kaplan-Meier curves in Figure 5B. The standard error and 95% confidence interval for these estimates is included. (DOCX) [file pone.0088955.s001.docx]

Table S1. Cases and events of patients with breast cancer, stratified by HIF-1α positivity.

| Total HIF-1α | Total N | N of Events | Censored | |
| --- | --- | --- | --- | --- |
|  |  |  | N | Percent |
| Negative | 61 | 18 | 43 | 70.5% |
| Positive | 86 | 35 | 51 | 59.3% |
| Overall | 147 | 53 | 94 | 63.9% |

Table S2. Mean survival of patients with breast cancer, stratified by HIF-1α positivity.

| Total HIF-1α | Mean Survival (years) | | | |
| --- | --- | --- | --- | --- |
|  | Estimate | Std. Error | 95% Confidence Interval | |
|  |  |  | Lower Bound | Upper Bound |
| Negative | 8.66 | 0.30 | 8.06 | 9.26 |
| Positive | 7.43 | 0.38 | 6.69 | 8.17 |
| Overall | 7.94 | 0.26 | 7.43 | 8.45 |

Table S3. Cases and events of patients with breast cancer, stratified by HIF-1α hydroxylation status.

| HIF-1α hydroxylation status | Total N | N of Events | Censored | |
| --- | --- | --- | --- | --- |
|  |  |  | N | Percent |
| HIF-1α -ve | 60 | 18 | 42 | 70.0% |
| HIF-1α +ve, HIF-OH -ve | 24 | 8 | 16 | 66.7% |
| HIF-1α +ve, HIF-OH +ve | 57 | 26 | 31 | 54.4% |
| Overall | 141 | 52 | 89 | 63.1% |

Table S4. Mean survival of patients with breast cancer, stratified by HIF-1α hydroxylation status.

| HIF-1α hydroxylation status | Mean survival (years) | | | |
| --- | --- | --- | --- | --- |
|  | Estimate | Std. Error | 95% Confidence Interval | |
|  |  |  | Lower Bound | Upper Bound |
| HIF-1α -ve | 8.63 | 0.31 | 8.03 | 9.24 |
| HIF-1α +ve, HIF-OH -ve | 7.99 | 0.64 | 6.73 | 9.25 |
| HIF-1α +ve, HIF-OH +ve | 7.07 | 0.48 | 6.12 | 8.01 |
| Overall | 7.89 | 0.27 | 7.37 | 8.41 |
